# Matters to address prior to introducing new life support technology in Japan: three serious ethical concerns related to the use of left ventricular assist devices as destination therapy and suggested policies to deal with them

**DOI:** 10.1186/s12910-018-0251-z

**Published:** 2018-02-27

**Authors:** Atsushi Asai, Sakiko Masaki, Taketoshi Okita, Aya Enzo, Yasuhiro Kadooka

**Affiliations:** 1 Department of Medical Ethics, Tohoku University Graduate School of Medicine, 2-1 Seiryo, Aoba-ku, Sendai, Miyagi 980-8575 Japan; 2 Department of Bioethics, Kumamoto University Graduate School of Medical Science, 1-1-1 Honjyo, Chuo-ku, Kumamoto, 860-8556 Japan; 3 Department of Bioethics, Kumamoto University Faculty of Life Sciences, 1-1-1 Honjyo, Chuo-ku, Kumamoto, 860-8556 Japan

**Keywords:** Japan, Destination therapy, LVAD deactivation, Advance directives, Advance care planning, Super-aging society, Culture

## Abstract

**Background:**

Destination therapy (DT) is the permanent implantation of a left ventricular assist device (LVAD) in patients with end-stage, severe heart failure who are ineligible for heart transplantation. DT improves both the quality of life and prognosis of patients with end-stage heart failure. However, there are also downsides to DT such as life-threatening complications and the potential for the patient to live beyond their desired length of life following such major complications. Because of deeply ingrained cultural and religious beliefs regarding death and the sanctity of life, Japanese society may not be ready to make changes needed to enable patients to have LVADs deactivated under certain circumstances to avoid needless suffering.

**Main text:**

Western ethical views that permit LVAD deactivation based mainly on respect for autonomy and dignity have not been accepted thus far in Japan and are unlikely to be accepted, given the current Japanese culture and traditional values. Some healthcare professionals might regard patients as ineligible for DT unless they have prepared advance directives. If this were to happen, the right to prepare an advance directive would instead become an obligation to do so. Furthermore, patient selection for DT poses another ethical issue. Given the predominant sanctity of life principle and lack of cost-consciousness regarding medical expenses, medically appropriate exclusion criteria would be ignored and DT could be applied to various patients, including very old patients, the demented, or even patients in persistent vegetative states, through on-site judgment.

**Conclusion:**

There is an urgent need for Japan to establish and enact a basic act for patient rights. The act should include: respect for a patient’s right to self-determination; the right to refuse unwanted treatment; the right to prepare legally binding advance directives; the right to decline to prepare such directives; and access to nationally insured healthcare. It should enable those concerned with patient care involving DT to seek ethical advice from ethics committees. Furthermore, it should state that healthcare professionals involved in the discontinuation of life support in a proper manner are immune to any legal action and that they have the right to conscientiously object to LVAD deactivation.

## Background

### Introduction of destination therapy in a Japanese clinical setting

Destination therapy (DT) is the permanent implantation of a left ventricular assist device (LVAD) in patients with end-stage, severe heart failure who are ineligible for heart transplantation. Since 2002, DT-LVAD has been covered by Medicare, a national health care plan in the United States [1, 2]. Japan is currently scheduled to conduct a clinical trial for DT use as a way to test the inclusion of DT as a treatment covered by national insurance that can be utilized by patients who are ineligible for heart transplantation [3, 4].

The use of DT would enable patients with end-stage heart failure to improve the quality of life (QOL, a multidimensional construct that includes performance and enjoyment of social roles, physical health, intellectual functioning, emotional state, and life satisfaction or well-being) and prognosis, and may even allow them to return to independent living [5]. Compared to maximal medical therapy, which yields a one-year mortality rate of 25–50%, implantable LVADs have been found to be superior, yielding one-year survival rates of 70–80% and two-year survival rates of 60–70% [6–10]. Relevant medical societies such as the Japanese Circulation Society and Japanese Society for Cardiovascular Surgery are proactively working to allow for expanded DT use and for this practice to be covered by national health insurance in Japan.

LVAD in DT is associated with many complications, including serious infection, thromboembolic events, cerebrovascular complications, postoperative bleeding, gastrointestinal hemorrhage, hemolysis, right heart failure, and aortic incompetency [8, 11, 12]. In particular, diagnoses such as infection/sepsis, stroke/neurologic issues accompanied by declining neurocognition, cancer, renal failure, multi-organ failure, impending pump failure or lack of candidacy for device exchange in cases of mechanical failure or thrombosis, and declining functional ability prompt patients and medical professionals to consider withdrawal of support [13].

In Japan, guidelines have been developed for DT patients in order to assist their end-stage decision making [10, 14]. Other highly relevant guidelines are also available [15, 16] and overlap in their perspectives on basic ethical ideas concerning end-of-life care. All suggest the importance of informed consent, utilization of an ethics review committee, multi-professional approaches, and the preparation of advance directives related to end-of-life treatments. That said, we are concerned that our conventional ethics may prevent the prudent use of this new medical technology, and worry that, despite great efforts taken to introduce DT into Japanese society, if current end-of-life judgments in Japan are not modified, these could hinder the beneficial use of DT.

Under the present circumstances, some patients who suffer from severe complications from DT use would end up dying a miserable death desired by no one because LVAD deactivation is not accepted, while other patients would be able to benefit from DT with prolonged life and a high QOL. Therefore, pressing issues here include the need for substantial revolution in Japanese medical ethics and preparation of ethical guidelines related to DT. However, to our knowledge, no published study to date has considered ethical arguments concerning the use of DT in Japan. In this paper, we first discuss basic ethical considerations related to DT practice. This will be followed by a discussion of serious ethical situations regarding LVAD deactivation, advance directives, and patient selection. Finally, we will propose countermeasures to implement in the near future so that patients, families, medical professionals, and societies can receive the maximum benefits of expanded DT use.

## Main Text

### Ethical considerations concerning destination therapy

Several ethical guidelines, considerations, and case studies related to DT have been published in Western countries [13, 17–23]. Most frequently, the relevant guidelines indicate that patients with sufficient decision-making capabilities should make their own decisions and provide consent concerning DT with a full understanding and appreciation of all treatment options. The guidelines typically include information on medical treatment and palliative care, the nature of DT, its influence on QOL, adverse events, and complications, as well as instructions for deactivation and device exchange in cases of mechanical failure or thrombosis. It is also recommended that patients prepare advance directives and designate a surrogate once they gain an understanding of the relevant end-of-life issues. In addition, it is crucial for all involved to conduct advance care planning (ACP) in the event of complications that would reasonably lead to deactivation of LVAD, so that patients can fully understand and appreciate the nature and consequences of the treatment [13, 17, 18, 23].

Chamsi-Pasha et al. reported that, when practicing DT, its LVAD deactivation can be the biggest ethical challenge. Therefore, every center which provides DT should have a process in place for discontinuing LVAD support, and all DT patients should have the option of deactivating DT when the burdens outweigh the benefits [18]. The ethics guidelines for DT, published in 2006, argue that grounds for ethical permissibility of LVAD deactivation exist when patients cannot appreciate the benefits of continued aggressive therapy and will only experience the burdens [23]. The study also indicated that competent, informed patients (or their surrogates) have the right to request the withdrawal of any life-sustaining intervention they perceive as excessively onerous relative to the benefits [18]. Beauchamp and Childress argued that physicians are not obliged to provide treatments they believe are futile to patients [24]. In cases of deactivation or withdrawal of life support, patients die of their underlying conditions and thus their death can be considered a natural death [13, 18]. The distinction between ‘killing’ and ‘letting die’ separates permissible from condemnable practice [24]. Of course, there are those who oppose these rationales [18–20, 25].

In our view, serious problems arise when a new technology such as DT is introduced because its proper application and termination must be dealt with first. The beneficial use of DT would require Japan to urgently adapt to new cultural norms and social attitudes towards end-of-life ethics, which differ significantly from traditional Japanese morals. Some of the aforementioned Western ethical opinions, especially those regarding the rationale for LVAD deactivation, have not been accepted thus far in Japan. Nor is it clear that they would be accepted in the future, given Japan’s current unstable legal situation. Even if relevant medical associations were to publish several guidelines and proclaim ethical norms with regard to DT use and deactivation, the operation of ethically appropriate DT is impossible unless social barriers toward the implementation of ethically favorable choices are eliminated, and unless all parties involved in DT are in full agreement with the relevant ethical principles and are motivated to respect them. Bruce et al. indicated that, with the introduction of any new medical technology, the diffusion of the technology and its proliferation often outpaces healthcare providers’ comfort levels [21]. If our socio-cultural attitude concerning medical ethics does not change, ethically problematic situations pertaining to LVAD deactivation, advance directives, and patient selection will most certainly follow.

### Serious ethical problems concerning destination therapy in Japan: 1. Deactivation

Given the current circumstances in Japan, deactivation of LVAD would be extremely difficult even if all parties including patients, their families, and medical professionals agreed with the request to do so. Some would claim that even if both the family and medical professionals are convinced that continuation of DT is pointless due to deterioration in the patient’s condition (e.g., severe renal failure, hepatic insufficiency, or serious cerebral infarction with no chance of recovery), the physician in charge has no legal authority to suspend it. In fact, under current Japanese legislation, when withdrawal of critical care leads to a patient’s death, the physician could be charged with murder. The fact that patients or their families have either allowed or requested deactivation of LVAD is irrelevant [26]. We argue that this accurately describes the present situation in Japan, in the sense that there is no legal framework for death with dignity.

Some have noted that, while brain death is recognized as human death only in the case of organ donors in Japan, if patients under DT become clinically brain dead but still retain circulation, a physician must wait for complete cardiac arrest with no chance of recovery [27]. A nationwide survey revealed that only 2% of physicians specializing in emergency medicine and intensive care would discontinue life support for brain-dead patients; in fact, many physicians fear that withdrawal of life support from brain-dead patients might constitute homicide, and that they would be charged with murder [28]. Despite this prevailing view, it was reported that a respirator had been withdrawn from an 83-year-old woman who experienced a serious cerebral infarction. However, this type of life support withdrawal is an extremely rare event in Japan [29].

Japan currently lacks written legal regulations concerning the termination of medical intervention, and thus healthcare professionals are left uncertain about which actions are forbidden. A patient’s right to refuse life-sustaining treatment (including withholding and withdrawal) has not been substantially warranted, and advance directives have not been legally enforceable [28]. Some have argued that patient dignity is in danger due to a national refusal to accept major bioethical principles such as respecting patient autonomy, serving the best interests of patients, doing no harm, and ensuring fairness at the end of life [30].

Patient refusal of treatment also causes quite a bit of turmoil among those concerned with patient care. In many cases, healthcare professionals attempt to persuade patients to change their minds. Two primary reasons seem to exist for this intolerance of treatment refusal. First, as we have argued in the past [30], many healthcare professionals tend to regard patient refusal of treatment as a psychological, rather than an ethical, issue. Physicians tend to think that patients who refuse recommended treatments must suffer from a mental illness, misunderstand relevant medical information, or have lost normal function temporarily in stressful situations. They assume that patients would never reject their recommendations if they possessed normal cognitive function. By perceiving refusal of treatment as a psychological problem, those concerned with patient care fail to grasp deeper problems regarding values such as liberty, privacy, and dignity.

The second reason is that some Japanese healthcare professionals, as well as patient families, favor interventions over doing nothing, even if this goes against the explicit informed refusal. When “doing something,” rather than evaluating beneficial outcomes for patients, becomes the goal in clinical settings, extraordinary measures such as DT are never considered useless or futile due to the unconscious benefit of alleviating regret in the physician and family [30].

However, there is also an increasing number of news reports of life sustaining treatments being withdrawn. Some televised news programs have even shown the actual act of withdrawing treatment [31]. After the guidelines of the Ministry of Health, Labour and Welfare mentioned later in this paper were enforced, there have been no cases of police involvement, and many legal scholars have confirmed the legality of withdrawing life-sustaining treatment [15].

### Serious ethical problems concerning destination therapy in Japan: 2. Advance directives

According to Japan’s guidelines for DT, when patients reach the terminal stage, the medical team should educate them sufficiently in order to obtain informed consent to provide no further treatment and to discontinue LVAD support. The guidelines also suggest that it is preferable for healthcare professionals to consult patients, their families, and caregivers in advance about appropriate countermeasures for clinical situations resulting in the end-of-life state [10]. In the same vein, another guideline recommends that patients and their families should fully understand terminal care concerning DT and that patients should prepare their advance directives regarding life-sustaining treatments for the terminal stage [14]. Other relevant guidelines advocate the importance of advance directives together with ACP [15, 16]. Recognition of and interest in ACP have grown and its usage is increasing rapidly in Japan [32]. Healthcare professionals have reportedly helped to promote patient preparation of their own advance directives in some medical facilities [33].

If DT becomes widely used as a treatment covered by health insurance, it is anticipated that healthcare professionals would request or even persuade their patients to prepare DT-related advance directives more often. We strongly believe that preparation of advance directives is important in order to live one’s final days with dignity, and that guidelines of Japan as well as Western countries are ethically appropriate. However, serious concerns remain about preparing advance directives in situations such as those mentioned above. First, patients and their families could be led semi-forcibly to present their advance directive. In addition, some medical professionals may not appreciate the essence of the right to self-determination or the importance of continuous communications among those concerned through ACP and just demand that patients and their families hand their advance directives in as a prerequisite condition to receive DT.

In other words, the concern is that some healthcare professionals will regard patients as ineligible for DT unless they have prepared advance directives. If this were to happen, then the right to prepare an advance directive would instead become an obligation to do so. One opinion not specific to DT is that a national campaign is required in which people prepare a brief note stating something such as “my expectations about healthcare when I am dying…,” because a large amount of medical expenses are incurred for unwanted life-sustaining interventions [34].

However, few people in Japan discuss advanced directives or wishes upon death, and this likely reflects their beliefs of *kegare* (impurity) and *kotodama* (miraculous power of language). The Shinto-based Japanese mentality considers and avoids death as *kegare* (impurity), it is taboo to think directly about death or dying for many Japanese people [35]. This faith is rooted in the cultural belief of *kotodama* (miraculous power of language); that is to say, once you have mentioned “death” vocally, the voice evokes death in reality [36].

Thus, several problems arise with the uniform imposition of advance directives on candidates for DT use. First, many Japanese patients are not eager to prepare advance directives. According to a nationwide poll by The Yomiuri (September 2013), 31% of respondents have discussed unwanted medical treatment when they reach the terminal stage with their families, whereas 68% have not. In addition, 44% of respondents expressed interest in preparing advance directives, 43% had no interest, and only 1 % had actually prepared for them [37]. The Ministry of Health, Labour and Welfare conference in 2013 reported that 2.8% of the population had discussed in detail with their family the treatment they would desire when they reach the final stage of their lives, while 56% have never discussed this matter [38]. Therefore, on the one hand, routine preparation of advance directives in clinical settings would be useful for patients who are willing to do so. On the other hand, significant psychological trauma may be experienced by both patients and their families if they do not desire to do so.

Another concern is that even if patients reluctantly prepare advance directives, including desires about treatment withdrawal at the end of life, it is highly likely that their wishes will not be fulfilled because some doctors would worry about the consequences of their acts (e.g., social blame and lawsuits) and not comply with those wishes. In that case, patients would have been forced to do unnecessary and useless work.

Finally, the utility of advance directives is limited in several ways including the following: contents of the directives may become unsuitable for the actual situation or too abstract to understand; families may oppose them; patients may be unable to envision the treatment choices in detail; patients do not wish to talk or think about the end stage and death; patients want to entrust others with these decisions; or there is not enough information needed to assess the likelihood of recovery due to medical uncertainties [32].

### Serious ethical problems concerning destination therapy in Japan: 3. Patient selection

Patient selection for DT poses yet another ethical issue. In the Japanese guidelines for DT, it is stated that patients with any of the following conditions are excluded from therapy: life expectancy of longer than 2 years due to heart failure, average life expectancy of less than 5 years due to the presence of other diseases, 65 years of age or older, a body mass index over 25, irreversible dysfunction in critical organs, any central nervous system diseases, any malignancies, or an inability to self-manage the device due to a brain disorder [10, 14]. The guidelines also suggest that it is preferable to have reliable caregivers either within the immediate family or other relatives available. When DT use is considered strictly from a medical perspective, these exclusion criteria are appropriate and ensure efficient utilization. However, the possibility that those guidelines may not be applied properly when it comes to patient selection in actual Japanese clinical settings is deeply disconcerting.

Our fear is that, if DT was to be widely used as an insurance-covered therapy in the near future in Japan, the exclusion criteria above would quickly become moot, and DT would be provided to patients who do not fulfill the criteria described in relevant guidelines. This is because many people tend to emphasize more heavily the sanctity of life (SOL) over QOL, and tend to commence life support and continue it regardless of patient values, clinical situation, or consciousness [30]. For example, if a patient is in a persistent vegetative state (PVS) and the family requests advanced medical treatment, it is not rare that such treatment would be provided. Even if the vegetative stage is expected, the family would likely not refuse intensive emergent care for the patient. As a result, treatment continuation for PVS patients becomes a huge burden on limited medical resources [39]. It is doubtful that a strong request for DT use from a chronic dialysis patient could be declined based on a guideline, given the general culture in which hemodialysis has been provided even for PVS patients and those with severe dementia. Therefore, we assume that the possibility of DT use for patients with serious complications other than severe heart failure (e.g., dementia) is not low.

Together with the SOL principle, a lack of cost-consciousness regarding medical expenses among Japanese people could also contribute to DT use among patients considered ineligible based on guideline criteria. Japanese people receive medical treatment under a universal health insurance system as a social security based on Article 25 (right to live) of the national constitution, and the subsidy system for any expensive medical treatment caps the upper limits on individual payment to minimize patient expenditure [40]. In view of this system, both healthcare providers and receivers in Japan are not very cost-conscious. Patients and their families would strongly demand the use of life-saving DT unless the cost was something they had to bear themselves. This is particularly true in situations for which DT use would guarantee the patient several years of independent living, while no DT use would imply certain death. Similarly, medical professionals may offer DT to patients whose conditions are too mild or too severe for optimal DT use according to relevant guidelines, in an attempt to save them.

Whether or not these aforementioned patient selection criteria constitute either ethical or social discrimination should also be considered. In Japan’s super-aging society, the justification for excluding patients for DT on the basis of their old age is questionable. Excluding patients over 65 years of age from the DT candidate pool is too arbitrary, and some would argue the wisdom in using age as a standard for health resource distribution. Controversy remains around the question of whether or not age-based selection is discrimination based on ageism or impartial distribution when deciding accessibility to treatment [2]. Unlike very scarce donated organs, the LVAD used for DT can be produced according to demand, and thus, putting an age limit according to rarity may not be justified. In Japan’s super-aging society, limiting DT use by establishing an upper limit of 65 or 70 years of age would become increasingly more difficult. Exceptions for its use will increase steadily, and unless the ethical validity of patient selection criteria is justified, society would not accept an age-based exclusion criterion.

## Conclusions

Given the current situation in Japan, we fear the following scenarios may ensue in future healthcare settings: DT is commenced for a patient over 80 years of age with severe dementia and no advance directives according to the family’s strong demands. Even if the patient’s medical condition were to worsen to the point that serious complications occurred, such as renal failure, bleeding, sepsis, and severe consciousness disturbance, no one would be able to withdraw DT or expensive life support treatments in the ICU, and these would continue endlessly until the patient’s death. Another possible scenario could involve a patient with decision-making capacities who considers the termination of DT use. The family may even agree with the patient’s wishes, but doctors and nurses might ignore such a request, thinking that the patient’s desire for DT withdrawal represents a clinical symptom of an unstable mental condition or that they could potentially be prosecuted. Doctors may even perform strong sedation of the patient, ignoring the patient’s expressed desires. The patient now has no choice but to continue with DT until the last minute, spending exorbitant amounts of national insurance funding.

These tragic situations could be avoided if several specific treatment goals (aside from simply maintaining life support) were clearly set, and the choice to deactivate DT accepted when achieving goals becomes difficult. These goals should include respect for individual dignity, maintaining high QOL, and serving the best interest of the patient. Treatment plans and patient selection must also be carefully thought out so that patients can achieve these goals. To avoid inappropriate life prolongation, advance directives should be prepared and respected. To the extent possible, an enabling environment should be created, allowing patients to prepare advance directives spontaneously, considering the possibility that they may lose their decision-making capacity at some point. How can these three steps be achieved?

First, healthcare professionals must re-acknowledge the importance of a patient’s decision and accept treatment refusals, including those to withhold or withdraw DT. It is essential for health professionals to remember whose best interest they are serving when providing medical treatment. Healthcare professionals are obligated to respect a patient’s basic expectations regarding medical care expressed in their advance directives. In addition, Japanese society as a whole is obligated to establish a system in which healthcare professionals are guaranteed immunity from blame or prosecution for withdrawing DT from patients out of respect for their advance directives.

Second, we believe it reasonable to ask patients who require DT to prepare themselves for the possibilities and vulnerabilities their future may present. In particular, they should think about preparing advance directives with regard to DT. If patients are unwilling to do so, then healthcare professionals in charge should explain the aim, role, utility, and limitations of advance directives and help patients understand their importance. Ideally, this would prompt patients to voluntarily prepare advance directives lest they are forced to do so under duress.

Patients need to understand that the utility of advance directives includes any or all of the following: it can help create a dignified and peaceful end of life; it can help a patient’s family and healthcare professionals in charge deliberate and decide the best course of action on the patient’s behalf; and it can prevent enormous amounts of money from being spent on unwanted life prolongation, which would be undesirable for all involved. Without a patient’s clearly expressed will, especially in present Japan, almost all individuals involved in decision-making concerning life support cannot help but continue life-prolongation for the patient in order to avoid being blamed or sued for homicide.

All individuals in society must not forget that our society often pays high healthcare costs for unwanted end-of-life interventions [33]. Thus, we believe it is not unethical to ask patients who require DT for survival to prepare themselves for their future and to consider financial demands on the domestic healthcare system as a result of their DT use. However, any coercion into writing advance directives is unacceptable and it should be made clear that preparing an advance directive is not an obligation. That said, some patients would be unable to retain a positive attitude if required to prepare advance directives, even having received an explanation and encouragement from various professionals. If a patient lacks any advance directives for end-of-life interventions including DT, then withdrawing DT should be acceptable in accordance with decisions of the hospital ethics committee that adequately takes into consideration family wishes, unacceptably low QOL, a patient’s inability to appreciate the benefits of DT, the futility that the patient’s medical goals cannot be achieved, and the necessity to protect patient dignity. Alternatively, physicians may request permission from the patient who cannot complete advance directive to make relevant decisions for the patient.

In summary, there is an urgent need for Japanese society to establish and enact a basic act for patient rights. The act should include: respect for a patient’s right to self-determination; the right to refuse unwanted treatment; the right to prepare legally binding advance directives; the right to decline to prepare such directives; and access to nationally insured healthcare. It also should enable those concerned with patient care involving DT to seek ethical advice from independent ethics committees. Furthermore, the act should explicitly state that healthcare professionals involved in the discontinuation of life support in a proper manner are immune to any legal action. In particular, it should make clear that withdrawal of life prolongation by physicians would not be considered murder under certain circumstances.

The act might also refer to a healthcare professional’s right to conscientious objection to LVAD deactivation. This is because some healthcare professionals would sincerely and conscientiously disagree with discontinuation of life support such as DT, especially in Japan where the SOL principle is dominant. Some have pointed out that to deactivate DT is an act that will require much courage on the part of the doctor [19]. Therefore, if involvement in LVAD deactivation damages honest healthcare professionals, it will be important to have a procedure in place that would find other professionals willing to respect the patient’s right in this regard.

We conclude by offering further clarification on our arguments. First, some might question whether it is justifiable for us to maintain the need to shift attitudes toward end-of-life care and advance directives among those concerned, particularly if millions of Japanese people prefer not to deal with advance directives given the deeply ingrained beliefs of *kegare* and *kotodama*. Our response would be that descriptive ethics differs from normative ethics and that *anticipated* situations caused by wide DT use cannot be used as justification to not change the public’s views, even if it conflicts with Japanese culture.

Moreover, although only a minority of Japanese people prefer to discuss end-of-life care in advance, relevant surveys have shown that those who desire aggressive life-prolonging treatments also belong to a minority [37, 38]. Despite this, the “dependency” mentality of Japanese people might lead one to believe that other individuals, such as one’s family or healthcare professionals in charge, should choose the best course of action for the patient, without the need for explicit communication via an advance directive or ACP. Japanese people reportedly expect others to consider what they need and unconsciously expect others to act in their best interest. This concept is referred to as *amae* (“dependence” in Japanese) [41]. It is also a predominantly Japanese concept to entrust important decisions to others, which may have its ideological origin in Buddhism [42].

Second, difficulties with changing the die-hard cultural attitudes of Japanese people should be touched on. Some may consider our views that people’s behavior will change merely through explaining the importance of advance directives and setting treatment goals to be idealistic. We can agree with this to some extent, and certainly suggest the implementation of more realistic solutions, such as the aforementioned legal changes, as well as large-scale efforts aimed at educating people on the advantages of advance directives for all concerned. In this regard, the Ministry of Health, Labour and Welfare has made efforts to promote ACP in clinical settings and advance preparations for one’s death [15]. The legality of withdrawing treatment in appropriate circumstances, as reflected in the recent cases discussed above, is also gaining traction, although there are still uncertainties. In order to develop a more reliable system, ethical support systems for end-of-life care decision making should be established in medical settings. We may also need to introduce limits on reimbursements for non-qualified candidates, with a careful review of records to ensure legitimate DT use.

Third, our arguments might give some the impression that we are against introducing DT, given the potential suffering of patients and financial drain on healthcare resources. This is certainly not our intent. Instead, we propose the wise use of DT and the need to reduce risks associated with its broad introduction. As technology for life support advances, corresponding advances must also occur in the ethics involved in order for the technology to be used wisely.

## Abbreviations

ACP: Advance care planning
DT: Destination therapy
LVAD: Left ventricular assist device
